# Unraveling the environmental and anthropogenic drivers of bacterial community changes in the Estuary of Bilbao and its tributaries

**DOI:** 10.1371/journal.pone.0178755

**Published:** 2017-06-08

**Authors:** Mikel Aguirre, David Abad, Aitor Albaina, Lauren Cralle, María Soledad Goñi-Urriza, Andone Estonba, Iratxe Zarraonaindia

**Affiliations:** 1 Department of Genetics, Physical Anthropology & Animal Physiology, Faculty of Science and Technology, University of the Basque Country (UPV/EHU), Leio, Spain; 2 Environmental Studies Centre (CEA), Vitoria-Gasteiz, 01008, Spain; 3 Department of Surgery, University of Chicago, 5841 S. Maryland Ave., Chicago, IL, United States of America; 4 Equipe Microbiologie et Environnement, IPREM, UMR CNRS 5254, Bâtiment IBEAS, Université de Pau et des Pays de l’Adour, BP1155, Pau, France; 5 IKERBASQUE, Basque Foundation for Science, Bilbao, Spain; Loyola University Chicago, UNITED STATES

## Abstract

In this study, 16S rRNA gene sequencing was used to characterize the changes in taxonomic composition and environmental factors significantly influencing bacterial community structure across an annual cycle in the Estuary of Bilbao as well as its tributaries. In spite of this estuary being small and characterized by a short residence time, the environmental factors most highly correlated with the bacterial community mirrored those reported to govern larger estuaries, specifically salinity and temperature. Additionally, bacterial community changes in the estuary appeared to vary with precipitation. For example, an increase in freshwater bacteria (*Comamonadaceae* and *Sphingobacteriaceae*) was observed in high precipitation periods compared to the predominately marine-like bacteria (*Rhodobacterales* and *Oceanospirillales*) that were found in low precipitation periods. Notably, we observed a significantly higher relative abundance of *Comamonadaceae* than previously described in other estuaries. Furthermore, anthropic factors could have an impact on this particular estuary’s bacterial community structure. For example, ecosystem changes related to the channelization of the estuary likely induced a low dissolved oxygen (DO) concentration, high temperature, and high chlorophyll concentration period in the inner euhaline water in summer (samples with salinity >30 ppt). Those samples were characterized by a high abundance of facultative anaerobes. For instance, OTUs classified as *Cryomorphaceae* and *Candidatus Aquiluna rubra* were negatively associated with DO concentration, while *Oleiphilaceae* was positively associated with DO concentration. Additionally, microorganisms related to biological treatment of wastewater (e.g *Bdellovibrio* and *Zoogloea)* were detected in the samples immediately downstream of the Bilbao Wastewater Treatment Plant (WWTP). There are several human activities planned in the region surrounding the Estuary of Bilbao (e.g. sediment draining, architectural changes, etc.) which will likely affect this ecosystem. Therefore, the addition of bacterial community profiling and diversity analysis into the estuary’s ongoing monitoring program would provide a more comprehensive view of the ecological status of the Estuary of Bilbao.

## Introduction

Estuaries are one of the most dynamic, complex, and species-rich ecosystems [1] primarily due to their tributaries’ discharges [2]. Nutrient input from tributaries convert estuaries into highly productive environments with tide-dynamics that cause a net movement of organic matter and other nutrients from the estuary to the sea [3]. Furthermore, physicochemical parameters—salinity, temperature, pH, and dissolved oxygen (DO) concentration—are highly variable within estuaries due to the effect of freshwater input from rivers [4] and generally show a defined seasonality [5–7].

Bacteria are highly sensitive to physicochemical fluctuations [8]. To elaborate, the microbial networks of an estuary are involved in multiple diverse physicochemical processes, such as the carbon and energy pathways whereby bacteria use energy in non-living detrital carbon stores to produce microbial biomass, the chelation of metal [9], and the various processes crucial to microbiological responses to pollution. Thus, analysis of bacteria could become an important component of biological monitoring programs for the evaluation of estuarine water quality [10,11].

Urban estuaries are typical targets for water monitoring, because they fall under considerable anthropogenic pressure [12] due to their proximity to cities and harbors. The Estuary of Bilbao, the focus of this study, has suffered structural modifications in that large-scale reclamation of intertidal areas reduced the original, expansive estuary to a simple tidal channel in the mid-19th century [12]. This channelization altered the water circulation and turnover patterns in the Estuary of Bilbao, which modified both the abiotic and biotic processes as well as the seasonal patterns in the plankton community [13]. Furthermore, since the beginning of the industrial age, wastes from the city and factories have impacted the Estuary of Bilbao’s ecosystem [14]. In spite of the estuary currently undergoing a recovery program that started in 1992 [14,15], there are still metal and hydrocarbon pollution remnants in the sediments and water. However, the reduction in polluting industries and the progressive implementation of an integrated sewage treatment plant have started the process to water quality [15]. In order to survey the changes in estuarine quality, a monitoring program that began in 1989 has been examining the physicochemical variables in the water and sediments, as well as observing the fish and phyto-zooplankton communities by traditional taxonomic methods [16,17]. Besides, bacteria populations respond rapidly in terms of diversity, physiology and functional characteristics to environmental changes, and thus, the evaluation of their diversity changes and community structure could be suitable to evaluate anthropic impact. In addition, they are the base of the food web (i.e. photosynthetic bacteria) [9] with phytoplankton [18], however, they have not been studied yet in this estuary and are not included in the monitoring program. New technological advances, such as the development of Next Generation Sequencing (NGS) and its application in environmental samples, have made it possible to address this lack of data on the microbial community in high resolution, bringing forth the opportunity to incorporate this information into monitoring programs in hopes of achieving a more comprehensive view of ecosystems.

The use of amplicon sequencing to depict the makeup of bacterial communities in urban estuaries has been primarily focused on analyzing large estuaries with a well-defined salinity gradient (mixed or partially-mixed) like that of the Mississippi River in the United States [19], Sydney in Australia [20], and Kalama in Greece [21]. Although these estuaries are composed of dissimilar communities, common patterns can be observed throughout that point to salinity and temperature as main variables defining bacterial community changes in these large estuaries. In addition, significant shifts in community structure and composition have been linked to several climatological causes (rainfalls, spring tides, etc.). However, small estuaries such as the Estuary of Bilbao are characterized by a notably shorter water residence time and salinity gradient compared to those of large estuaries. The estuary’s unique characteristics cause freshwater to flow through the upper water mass without mixing with the deeper saline waters, becoming brackish upon entering the sea. It had yet to be tested whether the environmental features governing bacterial changes in large estuaries are also main factors influencing the community of small estuaries.

In an attempt to define the key determinants that drive microbial ecology fluctuations in the Estuary of Bilbao, the present study characterized the bacterial communities along the salinity gradient of the small estuary and its tributaries through 16S rRNA amplicon sequencing. A longitudinal sampling and analysis of the bacterial community was conducted to unravel the ecosystems changes along an annual cycle. We differentiated the effects of seasonal periods on the estuarine bacterial community and oceanic upper layers. Specifically, we focused on bacterial changes in the inner zone of the estuary in the summer months, as previous studies have reported that the water is eutrophicated in that area during the summer due to the channelization of this estuary [13,15,22]. Moreover, in order to further expound the putative effect of additional anthropogenic pressure in the estuarine microbial communities, we analyzed the impact of the Wastewater Treatment Plant (WWTP) on the Galindo River—a tributary of the Estuary of Bilbao. To this effect, previous studies have reported that the biological reactors of WWTP allow the proliferation of certain bacteria that enable the degradation of compounds in the wastewater [23]. Those microorganisms can have a significant impact on estuarine bacterial communities [24,25]. In summary, this is the first study that surveys the microbiome of a small estuary once highly polluted by industrialization—the Estuary of Bilbao. The spatio-temporal analysis conducted in this estuary will allow elucidation of the environmental parameters most responsible for its microbial community shifts.

## Material and methods

No specific permissions were required for the collection of the water samples used in this research project, as the study area under observation is not subject to relevant conservation or protection legislation. The study did not involve endangered or protected species.

### Study area

The Estuary of Bilbao—the last track of the Nervion-Ibaizabal River—is a small macro-mesotidal system located on the Basque coast, north of the Iberian Peninsula, on the Cantabrian Sea coast (43°19'N 3°1'W). The Estuary of Bilbao crosses Bilbao's metropolitan zone (~1 million inhabitants) and the center of the Biscay industrial area. The estuary is a narrow (50–2980 m wide) and shallow (6–30 m deep) channel that is 20 kilometers long. It was one of the most contaminated regions in Europe until the late 1980’s and has since undergone a water recovery program in attempts to remedy the damage [15]. Except during short periods of increased river discharge, euhaline waters (salinity >30 ppt) dominate within the estuary [26]. The Estuary of Bilbao is partially mixed in the outer portions and highly stratified within the inner half—freshwater isolated in the upper layers while the bottom layers remain euhaline [22]. This stratification is a consequence of channeling the original estuary [12], which caused the freshwater to begin flowing solely through the upper stratum bypassing the bottom saline water.

The Estuary of Bilbao has several tributaries: Nervion-Ibaizabal, Kadagua, Asua, Galindo, and Gobela. These tributaries flow through a variety of areas with diverse environmental characteristics. Among them, the Nervion-Ibaizabal River has the highest discharge into the estuary, representing 66% of the estuarine waters and a 1.900 square-kilometer basin. Of note, the Galindo River is exposed to a Waste Water Treatment Plant (WWTP) and the metallurgical industry, thus input from those establishments influence the entire ecosystem. The WWTP has an average daily effluent flow of 4,000 l / sec (According to the data provided by the WWTP, http://www.bizkaia21.eus/atalak/TerritorioSostenible/Lugares/datos.asp?id=3&IdPagina=36&idioma=ca). The Galindo river is a low flowing river, its discharge never reaches more than 3,000 l / sec (except in large rainfalls, [13]). In summer its discharge barely reaches 500 l / sec. For this reason, most of the Galindo river water can have its origin in the WWTP.

### Sample collection and physical and chemical analyses

Tributary samples were collected during the months of April, August and October 2014. A total of 12 samples (including two replicates) were collected in the last stretch of each tributary and from fixed points (primarily at bridges), avoiding areas affected by the tide. In the case of the Galindo River, the sampling station was 5 meters from the outlet of the WWTP.

**Fig 1:**
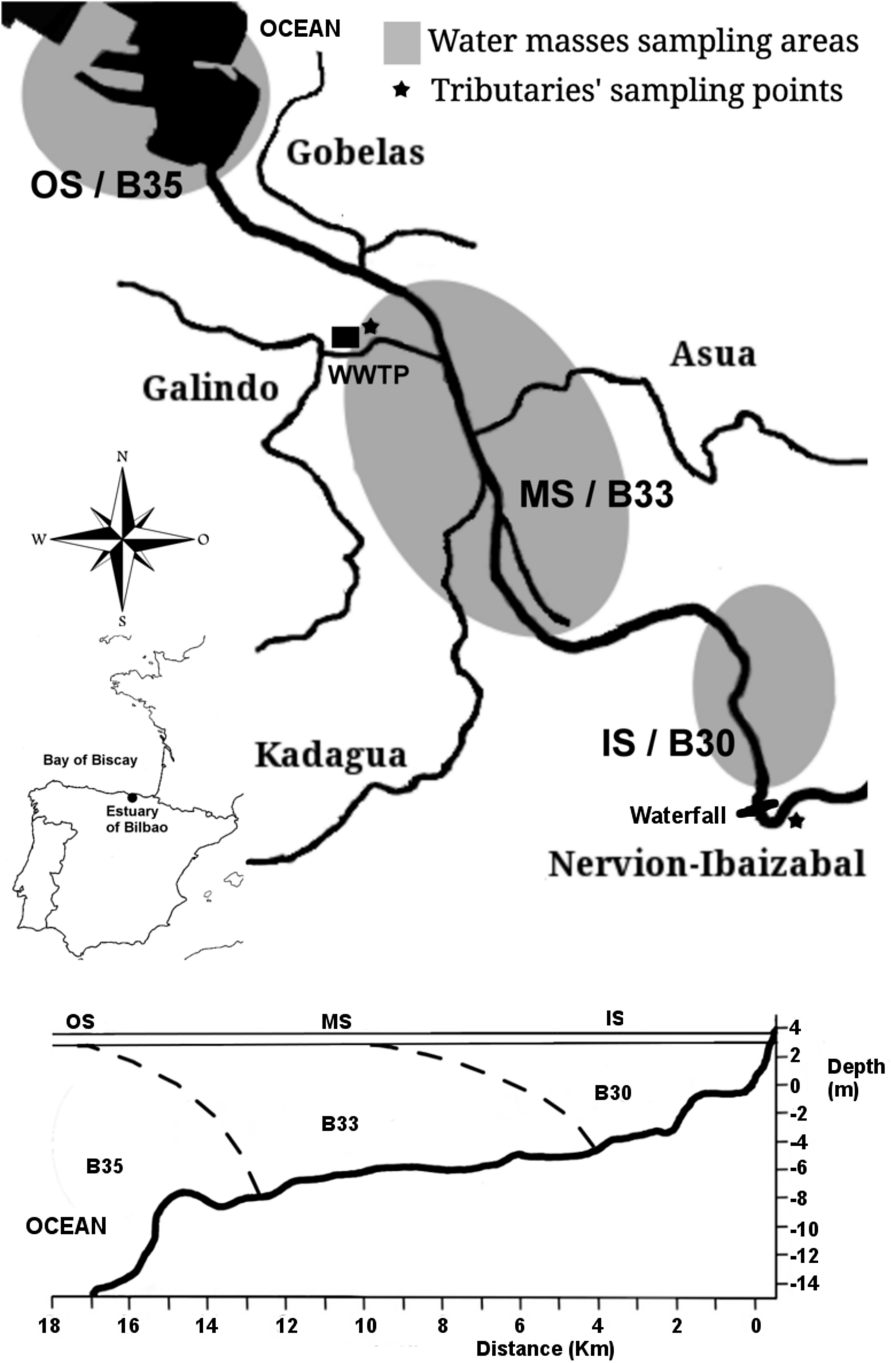
Map of the Estuary of Bilbao and its tributaries. Samples were collected at two tributaries in April, August, and October 2014 [Nervion-Ibaizabal (NER) and Galindo (GAL)] indicated by stars in the figure. In addition, samples were collected at the estuary, each month from August 2013 to October 2014, indicated in grey.

For the estuary samples, collection was carried out monthly from August 2013 to October 2014. In total, 171 samples (including two replicates) were collected for the 14-month period. Sampling took place only on days of neap tide coefficient (30–50), always at high tide, and at approximately the same time of day (10:00 A.M.-12:00 P.M.) to eliminate confounding variables. Salinity gradient points of 30, 33 and 35 ppt were localized along the estuary each month. Once the water mass stabilized, samples were collected at a middle depth (>3 m), below the halocline (B30, B33, B35), and at the upper layer of each euhaline water mass (surface samples: IS, MS, OS, respectively) (Fig 1). These six types of samples were collected at the estuary: 1) samples collected at salinity 30 ppt water mass. These waters are located in the inner zone of the estuary B30; 2) samples collected at the surface of B30, called IS for Inner Surface; 3) Samples collected at salinity 33 ppt, typically located in the intermediate zone of the estuary B33; 4) samples collected at the surface of B33, called MS for Middle Surface; 5) Samples collected at salinity 35 ppt, typically located in the outer most edges of the estuary B35; 6) samples collected at the surface of B35, called OS for Outer Surface (Fig 1).

Samples were collected using an oceanographic Niskin bottle. The water (10 L approx.) was stored in opaque plastic jerry cans in the field. Once in the laboratory, the water was filtered (5 L approx.) through 20 μNylon net filters (Millipore, 90 mm diameter) and bacteria were collected with 0.22 μm Durapore® membrane filters (Millipore, 47 mm diameter). Filtration was performed in triplicate using a Kitasato Flask and a vacuum pump. The whole process, from sampling to storage, took less than 3 hours to perform. All filters were store at -80°C until DNA extraction.

At each sampling point vertical profiles (every 0.5 m) of salinity, temperature, pH, and dissolved oxygen (DO) saturation (%) were obtained in situ using a YSI 556 MPS Multiparameter Probe. Water transparency was measured with a Secchi Disk. Chl-a concentrations were calculated from spectrophotometric measurements on acetone extracts using a monochromatic method with acidification [27]. In addition, precipitation data was obtained through the Hydrometeorology Service of the Regional Council of Bizkaia (http://www.bizkaia.eus/Ingurugiroa_Lurraldea/Hidrologia_Ac/Datos_meteo.asp?Idioma=CA&Tem_Codigo=2679).

### DNA extraction

Complete genomic DNA was extracted from the half of the 0.22 μm Durapore® membrane filters using PowerSoil DNA isolation kit (Mo Bio laboratories, Inc., Carlsbad, CA, USA) following the manufacturer protocol. The DNA quantity and quality of each sample was assessed by either a ND-1000 spectrophotometer (NanoDrop) or Qubit fluorimeter (Life technologies). To avoid cross-contamination all tools were flame-sterilized between samples and lab surfaces were decontaminated with DNA-ExitusPlus (Applychem) after each session. Finally, the DNA extractions were stored at -20°C until DNA sequencing.

### 16s rRNA amplification and sequencing

The 16S rRNA samples were amplified and sequenced by the Next Generation Sequencing Core at Argonne National Laboratory, Lemont, IL (USA) (http://www.earthmicrobiome.org/). Earth Microbiome Project's [28] protocols [29] were followed for the amplification and sequencing of the community 16S v4 region by using 515f and 806r primers that contained 12 bp barcodes for sequencing. The sequencing was carried out in two MiSeq runs (2x150 paired-end). The data is available in the QIITA portal (ID 10470) and on the ENA database (study: PRJEB14094).

### Bioinformatic pipeline

The raw sequences were trimmed using Sickle tool (v1.33) [30] with default parameters (including Phred score ≥ 20). Next, Pear software (v0.9.6) [31] was used to merge Illumina paired-end reads, using a cut-off of 0.01 (P-value) for the observed expected alignment score. Next, we utilized fastq-barcode.pl [32] to remove non-existent barcodes from the fastq achieved by Pear. Before carrying out the taxonomic assignment, the chimera sequences were removed by identify_chimeric_seqs.py in QIIME using the usearch61 (v7.0.1090) de novo method [33,34]. The resulting dataset was then analyzed by QIIME software (v1.9: [35]). We only included sequences that were 240–260 bp in length (average 253bp) to avoid background noise in the subsequent analyses. An open reference OTU picking method was used in QIIME for clustering using a 97% similarity cut-off using UCLUST algorithm (v1.2.22q) [33] and the taxonomy of the reference sequences was assigned based on Silva 119 database version [36] (clustered at 97% identity). The OTUs with which representative sequences failed in PYNAST alignment were discarded. After the taxonomical assignment, all chloroplast were removed from the BIOM file using filter_taxa_from_otu_table.py script in QIIME. Afterwards, samples with less than 5000 sequences were eliminated. Then, all OTUs with less than 10 sequences were removed. Finally, the BIOM file was normalized using metagenomeSeq’s CSS algorithm, which normalized sequences using the cumulative sum scaling transformation [37].

### Community composition analysis

First, alpha diversity analysis and taxonomic assignment were conducted to determine the community richness and composition. The alpha diversity (observed OTUs and Shannon) of the samples were calculated using phyloseq (v1.14) R package [38]. To visualize the bacterial community composition of the samples, taxa_summary_through_plot.py command in QIIME v1.9 software [35] was used.

In order to determine water mass specific OTUs as well as the OTUs that were present in all water masses, the core microbiome was analysed using compute_core_microbiome.py command on QIIME v1.9 [35]. The core microbial communities were defined as the OTUs that were present in 100% of the samples within each water mass (IS, MS, OS, B30, B33, B35) throughout the year. To compare the shared OTUs between the different water masses, a Venn diagram tool was used (http://bioinformatics.psb.ugent.be/webtools/Venn/).

A supervised learning analysis was performed for estuarine water masses using the Random Forests classifier, ten-fold cross-validation models, [39,40] and 1,000 trees. OTUs were considered “predictors” and sample type or water mass were the “class label”. This method determines the diagnostic power of bacterial profiles for predicting the characteristic community of the water masses by using a subset of samples to train a model that identifies unique features within data categories. The technique then determines the accuracy of the model by categorizing sample subsets that were not used to build the model. Through this method, we were able to evaluate not only the discriminative power in the microbial community to distinguish those groupings (sample type and water mass) but also the robustness of the groupings themselves. Therefore, the discriminative power of the microbial community in each water mass and the robustness of the groupings themselves were both evaluated for accuracy. Analysis of Similarity (ANOSIM) statistics (999 permutations) were carried out with the ANOSIM function [41] and were used to test whether grouping samples by salinity was significant.

Principal coordinate analysis (PCoA) plots [42] were used to examine community dissimilarity and determine the impact of environmental factors (salinity, temperature, pH, DO concentration, precipitation, Chl-a) on microbial community structure. Result visualizations were made using EMPeror tool [43]. Beta diversity was estimated using the unweighted UniFrac metric for 16S rRNA amplicon data. Also, an Unweighted Pair Group Method with Arithmetic mean (UPGMA) was used to construct a tree from the unweighted UniFrac beta diversity distance matrix. This analysis aimed to characterize the differences in phylogenetic community structure.

To calculate correlations between OTUs abundances and environmental parameters, Spearman's rank correlation coefficient (rho) was carried out, by which it was possible to identify which OTUs were related to different environmental variables—salinity, temperature, pH, DO concentration, water turbidity, precipitation and chlorophyll. The impact of these environmental factors on bacterial communities was analyzed using the bio-env method of vegan (v. 2.3–4) R package [41]. This method generates Spearman’s rank correlations between the community distance matrix and an euclidean environmental distance matrix and then ranks all environmental variables by their importance. In order to calculate the percentage of beta diversity variation in each water mass explained by precipitation, an analysis of Adonis was performed.

Finally, to understand the bacterial dynamics in the inner euhaline zone of the Estuary of Bilbao, where the low DO concentrations and high values of temperature and chlorophyll concentrations dominate in summer, we used extended Local Similarity Analysis (eLSA) software [44]. The analysis was performed using OTUs with highest abundance values at B30 samples. Following eLSA software guidelines, a total of 85 OTUs were included in the analysis. To adapt to the algorithm limitations and minimize computational cost, eLSA was used to reveal statistically significant local and potentially time-delayed association patterns between OTUs and environmental factors. Normalization of variables was performed by ‘robustZ’ method, including 14 time spots for the total number of sampling months. The rest of the analysis settings were set to default. Lastly, q-values were calculated to determine false-discovery rates. Correlations with q<0.01 were visualized in Cytoscape v3.2.1 [45], creating a continuous mapping-based network.

To identify the differences in OTU composition between water masses, a Kruskal-Wallis non-parametric test was carried out between tributaries and estuarine water masses. In this way, the OTUs whose abundances significantly differed between water masses were identified. Moreover, the community dissimilarity within the estuary and its tributaries were determined using a Detrended Correspondence Analysis (DCA) carried out by phyloseq (v. 1.14) R package [38].

## Results

The physico-chemical analysis showed that each of the observed water masses had individual dynamics throughout the year. For instance, euhaline masses (B30, B33, B35) show little annual variation in their physico-chemical parameters, while surface waters located in the lower half of the estuary (MS, OS) showed high variability (Table 1 and S1 Table). Furthermore, the inner euhaline zone (B30) was characterized by high turbidity and low DO saturation (Table 1). When analyzing the interaction among physicochemical factors, a negative correlation between temperature and precipitation was observed for all water masses (S1 File). In addition, temperature and salinity were negatively correlated at surface waters in the lower half of the estuary (MS, OS) while a negative correlation between temperature and DO saturation was evidenced in B30 (S1 File).

**Table 1:** Annual mean and standard deviation values of physico-chemical parameters for each water mass.

Water Mass (N° samples)	1/Turbidity (m)	Salinity (ppt)	Temp (°C)	DO (%)	pH	ChlA (ug/l)
**IS (N = 14)**	NA	1.085 ± 1.496	14.757 ± 4.841	100.91 ± 12.657	8.157 ± 0.109	NA
**MS (N = 14)**	NA	6.785 ± 9.755	15.306 ± 5.178	99.552 ± 17.418	8.086 ± 0.107	NA
**OS (N = 12)**	NA	28.17 ± 7.675	16.457 ± 3.648	115.283 ± 30.831	8.035 ± 0.146	NA
**B30 (N = 14)**	0.919 ± 0.779	29.905 ± 0.372	16.406 ± 3.926	36.454 ± 27.921	7.872 ± 0.152	1.331 ± 4.821
**B33 (N = 14)**	1.347 ± 0.709	32.929 ± 0.225	16.347 ± 3.553	87.467 ± 10.588	8.01 ± 0.082	1.341 ± 1.564
**B35 (N = 12)**	3.903 ± 2.220	34.781 ± 0.306	17.073 ± 2.966	104.773 ± 3.936	8.091 ± 0.102	0.910 ± 1.720
**NER (N = 3)**	NA	0.347 ± 0.081	17.877 ± 3.784	99.367 ± 3.383	8.203 ± 0.182	NA
**GAL (N = 3)**	NA	8.423 ± 12.033	20.493 ±± 2.353	76.3 ± 12.692	7.59 ± 0.560	NA

The annual values represented in the table were calculated based on the monthly measurements. Raw data are detailed in the S1 Table. The environmental parameters measured are: Turbidity (measured by Secchi disk depth), Salinity, Temperature (°C), Dissolved Oxygen (DO) saturation, pH, and Chlorophyll (ChlA).

**Fig 2:**
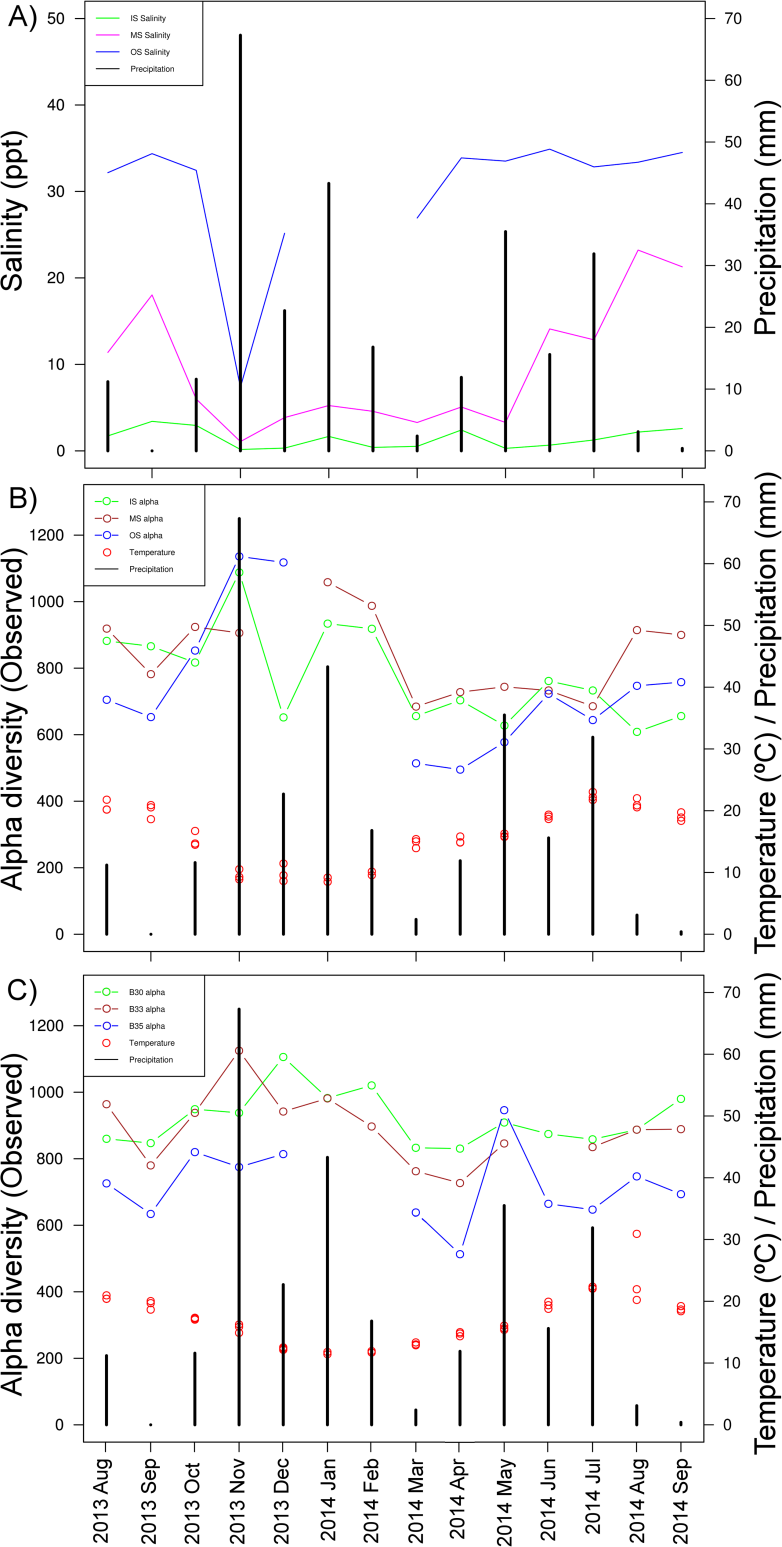
Main environmental features variations (temperature, salinity and precipitation) and community richness changes along the annual cycle. A) Monthly salinity and precipitation variation per surface water mass IS, MS, OS; B) Observed alpha diversity, temperature and precipitation fluctuation per surface water mass IS, MS, OS; C) Observed alpha diversity, temperature and precipitation changes per euhaline water mass B30, B33, B35.

With regard to bacterial community analysis, the high-throughput sequencing approach yielded a total number of 3.98 millions of 16S rRNA sequences in the 155 samples collected in this study after eliminating OTUs assigned to chloroplasts (13% of the reads), ones appearing in less than 10 reads, and samples with less than 5000 reads. The alpha diversity values of each water mass oscillate throughout the year. The major diversity changes were observed in the surface waters (IS, MS, OS) in the dates with greater precipitations (Fig 2). Precipitation causes the surface waters of the lower half of the estuary (MS and OS) to become brackish (5–30 ppt) from December to May, which increases community richness (Fig 2).

**Fig 3:**
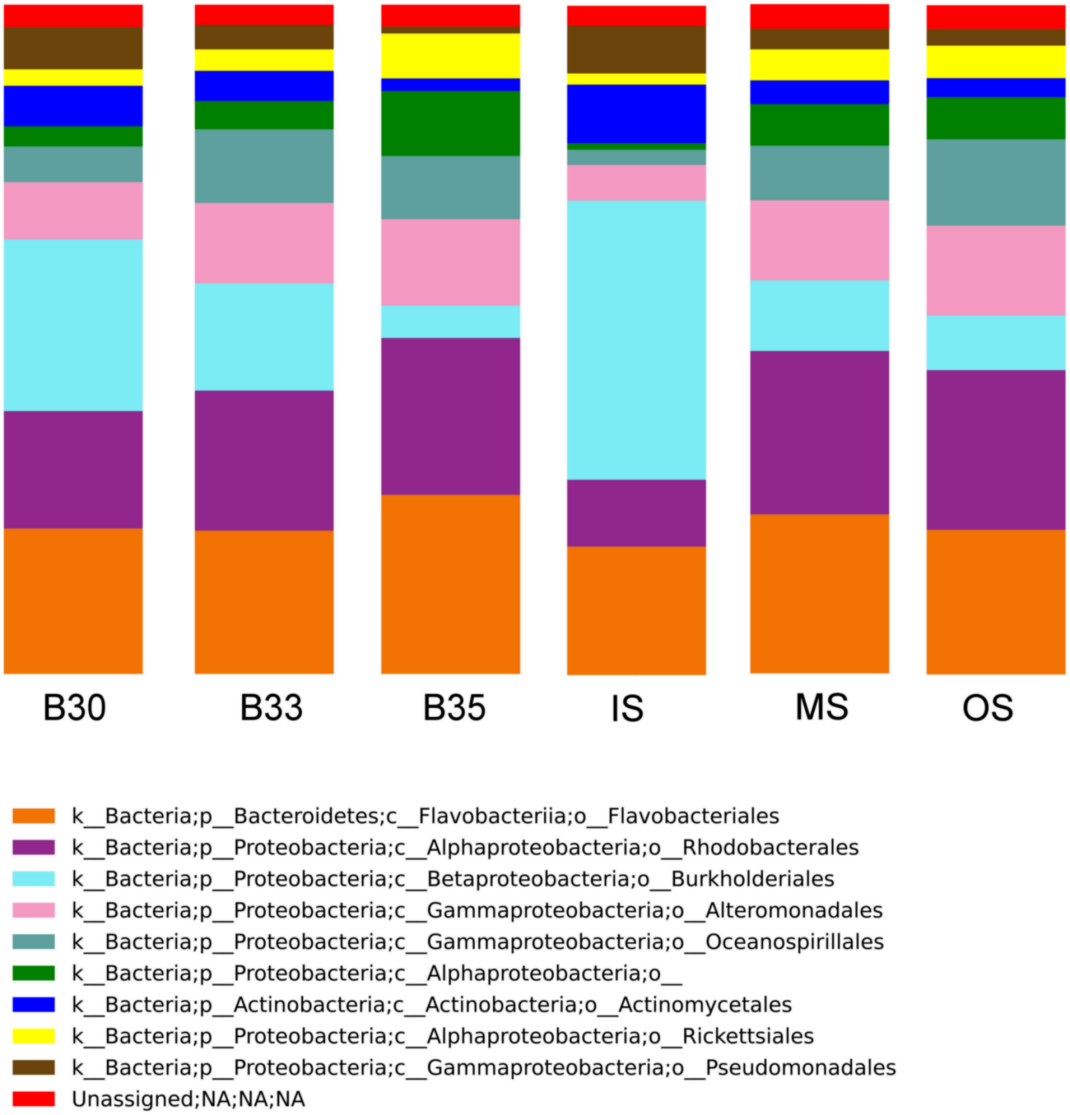
Microbial community composition in the water masses of the Estuary of Bilbao. OTUs relative abundances per water mass were plotted. Each column shows the mean relative abundance of the top 10 most abundant orders per water sample (B30, B33, B35, IS, MS, OS) along the annual cycle (14 months). These bacteria account for the 68% of the total community.

All euhaline water masses (B30, B33 and B35) were dominated by OTUs affiliated with *Flavobacteriales* (14.3–19.6%), *Rhodobacterale*s (14–17.1%), *Alteromonadale*s (8–9.4%), *Oceanospirillales* (7.4%), and *Burkholderiales* (7%) orders (Fig 3). Nevertheless, small but significant differences were found between euhaline samples (Anosim, R < 0.19, p < 0.034). In contrast, significantly high Anosim R-values were evidenced between surface waters (IS *vs*. OS, R = 0.785, p < 0.001). Within surface waters (IS, MS and OS), high abundances of OTUs related to *Flavobacteriales* (11.6–17.5%), *Rhodobacterales* (6–18%), *Actinomycetales* (5.3%), and *Pseudomonadales* (4.2%) were found. The *Burkholderiales* group was particularly abundant in IS (24.8%), decreasing gradually to reach a mean of 7.7% in OS (Fig 3 and S2 File). Surface waters were also characterized by high abundances of OTUs related to *Flavobacteriales* (11.6–17.5%), *Rhodobacterales* (6–18%), *Actinomycetales* (5.3%), and *Pseudomonadales* (4.2%).

The core OTU analysis (OTUs present in all the samples within each water mass during the entire cycle) showed that the euhaline samples contained the highest number of core OTUs: 94, 100 and 92 OTUs for the B30, B33 and B35 samples, respectively. Among the surface waters, IS showed the highest numbers of core OTUs (89), while the other two surface waters—MS and OS—showed fewer core OTUs (42 and 25, respectively). When analyzing the core OTUs shared amongst water masses, yearlong trends showed that the euhaline waters had a high number of shared OTUs between them (20) (S3 File). Interestingly, IS had the highest number of unique OTUs (50) while MS and OS had the lowest unique OTUs values (6 and 0, respectively). Random Forests model results evidenced a high classification error for MS and OS samples (0.35 and 0.6667 class error, respectively). Conversely, samples from IS, B30, B33 and B35 had a lower than 0.12 classification error (Table 2).

**Table 2:** Confusion matrix for the water masses of the Estuary of Bilbao.

	Assigned to						
Origin from	IS	MS	OS	B30	B33	B35	Class error
IS	20	1	0	1	0	0	0.0909
MS	3	13	1	2	1	0	0.35
OS	1	1	5	1	2	5	0.6667
B30	0	0	0	21	2	0	0.087
B33	0	0	0	1	23	0	0.0417
B35	0	0	0	1	1	15	0.1176

A Random forests classification analysis was conducted based on the communities dissimilarities among water masses. In this confusion matrix, the first column refers to where the samples were collected, while row numbers indicate the number of samples that are predicted to belong to each water mass. The classification error value is the rate of misclassified samples within each mass.

### Annual dynamics of bacterial communities

**Fig 4:**
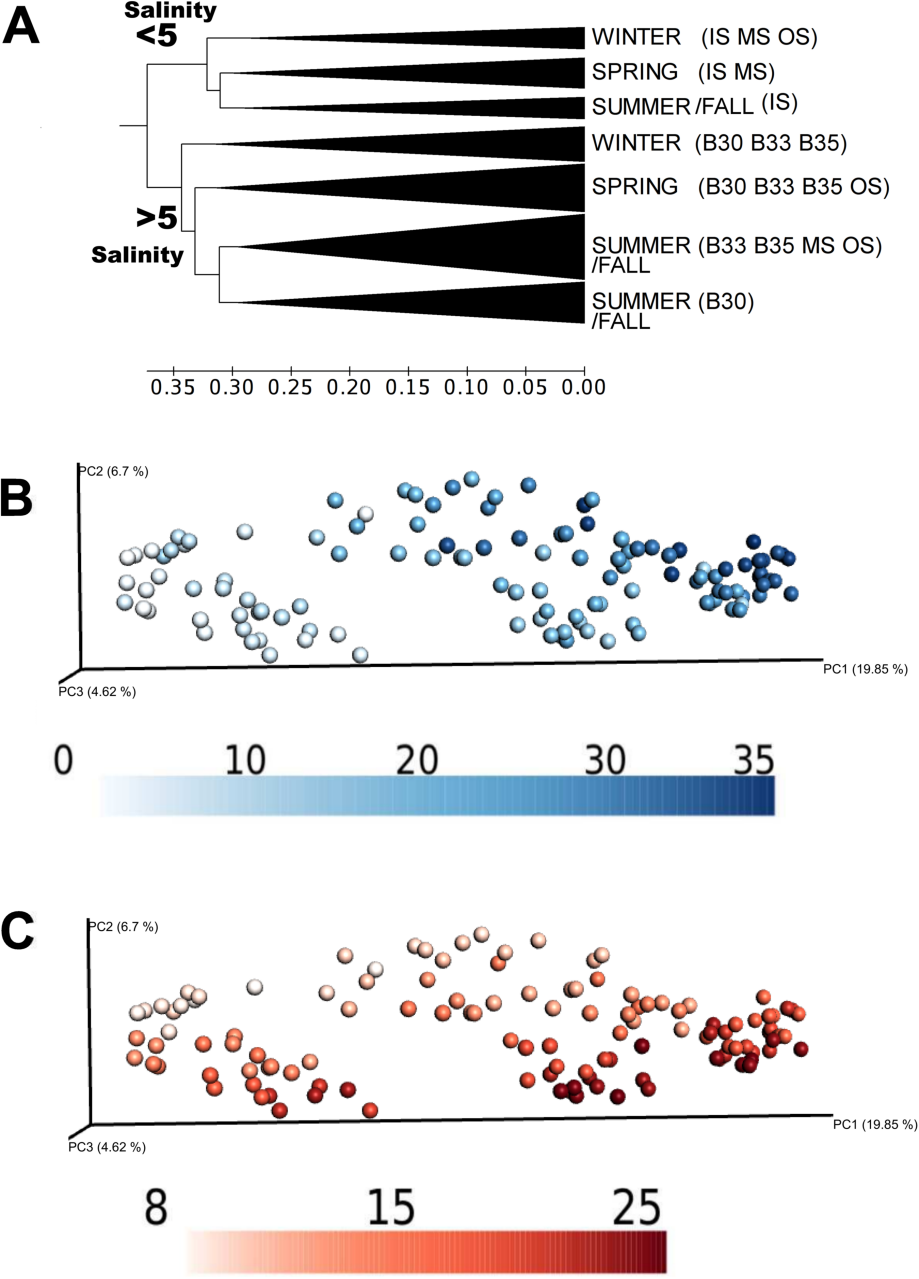
Dynamics and classification of bacterial communities of the Estuary of Bilbao. A) UPGMA tree of the samples from the Estuary of Bilbao based on the unweighted UniFrac beta diversity distance matrix. Samples with a beta diversity distance less than 0.30 are collapsed into same branches. Seasons are defined according to the natural temporal changes in the northern hemisphere, considering winter (22 Dec-21 Mar), spring (22 Mar-21 Jun), summer (22 Jun-21 Sept) and fall (22 Sept-21 Dec). B) Unweighted UniFrac distance principal coordinate analysis (PCoA plot) colored by salinity gradient: from freshwater (0 ppt) to saline water (35 ppt). Darker signifies a higher salinity. C) Unweighted UniFrac PCoA plot colored by temperature gradient: from low temperature (from 8°C) to high temperature (to 25°C). Darker signifies a higher temperature.

The UPGMA cluster method grouped the samples by salinity values; freshwater and saline water samples (brackish and euhaline, respectively). This result was also supported by the Spearman's correlation test, in which salinity had the highest values (rho = 0.719). Samples collected at salinities less than 5 ppt were differentiated from the rest with a beta diversity greater than 0.35 (Fig 4). Particular taxa were found to be significantly associated to the salinity gradient (S2 Table). Spearman's analysis related some bacterial families (*Comamonadaceae*, *Oxalobacteraceae*, and *Rhodocyclaceae*) with low saline concentration waters. In the same way, the taxa related with euhaline water masses such as *Halomonadaceae*, *Piscirickettsiaceae*, and *Pelagibacteraceae* were more abundant in the B35 water mass.

Additionally, season was a secondary driver of microbiome fluctuations, whereby samples were clustered into three seasons in the UPGMA tree: winter, spring, and summer/fall with a beta diversity value greater than 0.3 (Fig 4A). Similarly, temperature was the second strongest environmental factor varying in synchronously with bacterial community changes (Spearman rho = 0.342). This environmental variable showed a stronger correlation with surface waters communities (IS, OS, MS; rho > 0.57) than with euhaline ones (B30, B33, B35; rho < 0.46) (S3 Table). Certain OTUs were negatively related to temperature changes (*Pseudomonadaceae* and *Sphingobacteriaceae*) while other members showed a positive correlation (*Verrumicrobiaceae* and *Microbacteriaceae*) (S4 Table).

Beyond temperature, precipitation higher in winter and spring (S1 Table), seemed to reflect variations in the bacterial community makeup. The Spearman rank correlation evidenced that this feature correlation was higher for surface waters (IS, MS and OS) than for euhaline samples (S3 Table). In addition, a correlation gradient from the inner to outermost surface waters was found (rho = 0.20–0.36 in IS and OS, respectively). In rainy periods (December to May), MS and OS samples cluster within freshwater group (Fig 4A). The composition of MS and OS in that time has an increase of freshwater bacteria (S2 File) such as *Burkholderiales* order, mostly *Comamonadaceae* family, and *Pseudomonadales* (S2 File). Conversely, in low precipitation periods, MS and OS grouped together with euhaline samples communities in the UPGMA tree (Fig 4A).

**Fig 5:**
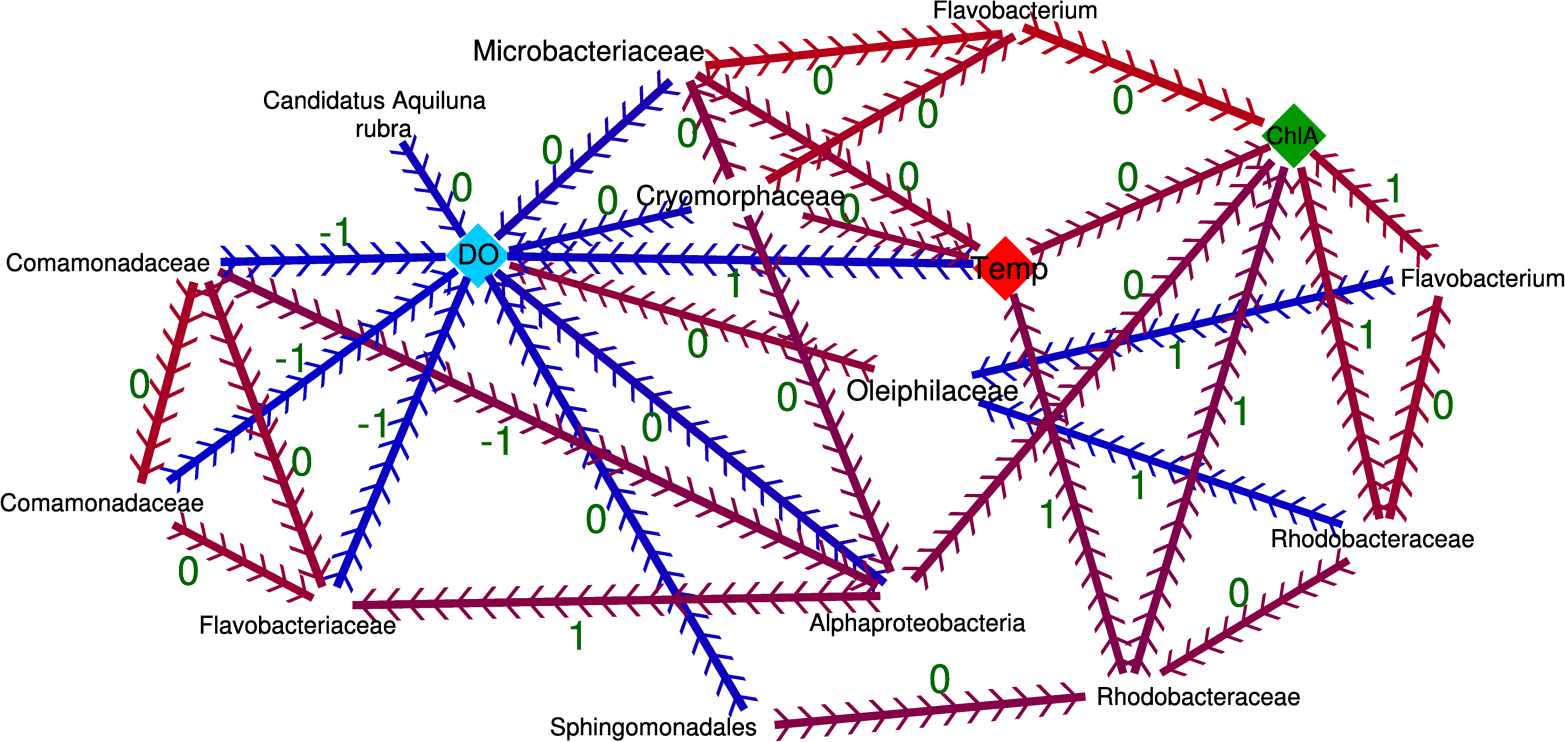
The OTUs significantly related with DO concentration, temperature, and chlorophyll concentration in B30 water mass through time. eLSA analysis was conducted for the 14 time points (total sampling months in duplicate) of the B30 water mass samples. In analysis, the 85 most abundant OTUs and all the environmental features measured were included (salinity, temperature, precipitation, pH, turbidity, and chlorophyll and DO concentration). The matrix of the variables was normalized by ‘robustZ’ method. A network was created with Cytoscape software [45] using the significant (q < 0.01) correlations obtained in the eLSA analysis. The directionality of the relationship is marked with arrows with its temporal delay (in months) in the edge label (green) and the relation type between them positive (red) or negative (blue).

Among euhaline samples, B30 water mass community clustered separately from the rest in summer (Fig 4A). The B30 water mass was characterized by high turbidity, acidic pH, high chlorophyll concentration, and low DO concentration (Table 1). For this water mass, DO concentration combined with temperature showed a rho explanation value of 0.809. According to the extended Local Similarity Analysis (eLSA), some OTUs were related to DO concentration, temperature and chlorophyll concentration through time (Fig 5), while no significant correlations were found for the remaining environmental variables (salinity, pH, turbidity, precipitation). *Alphaproteobacteria*, *Candidatus Aquiluna rubra*, *Comamonadaceae*, *Cryomorphaceae*, *Flavobacteriaceae*, *Microbacteriaceae*, and *Sphingomonadales* were negatively correlated with DO concentration. Specifically, *Comamonadaceae* and *Flavobacteriaceae* had a temporal delay of one month regarding DO concentration. Additionally, *Oleiphilaceae* positively correlated with DO concentration. The analysis showed that temperature and chlorophyll concentration were positively related to each other, while temperature was negatively related to DO concentration. This is also evidenced in the interaction of OTUs and environmental features, where the OTUs classified as *Rhodobacteraceae* and *Flavobacteriaceae* clades were positively related with temperature and chlorophyll concentration but not with DO concentration. Conversely, *Cryomorphaceae* and *Microbacteriaceae* were positively related to temperature but negatively with DO concentration (Fig 5). Even though, it was not possible to assign some of the significantly related OTUs further than family rank.

### Tributaries

**Fig 6:**
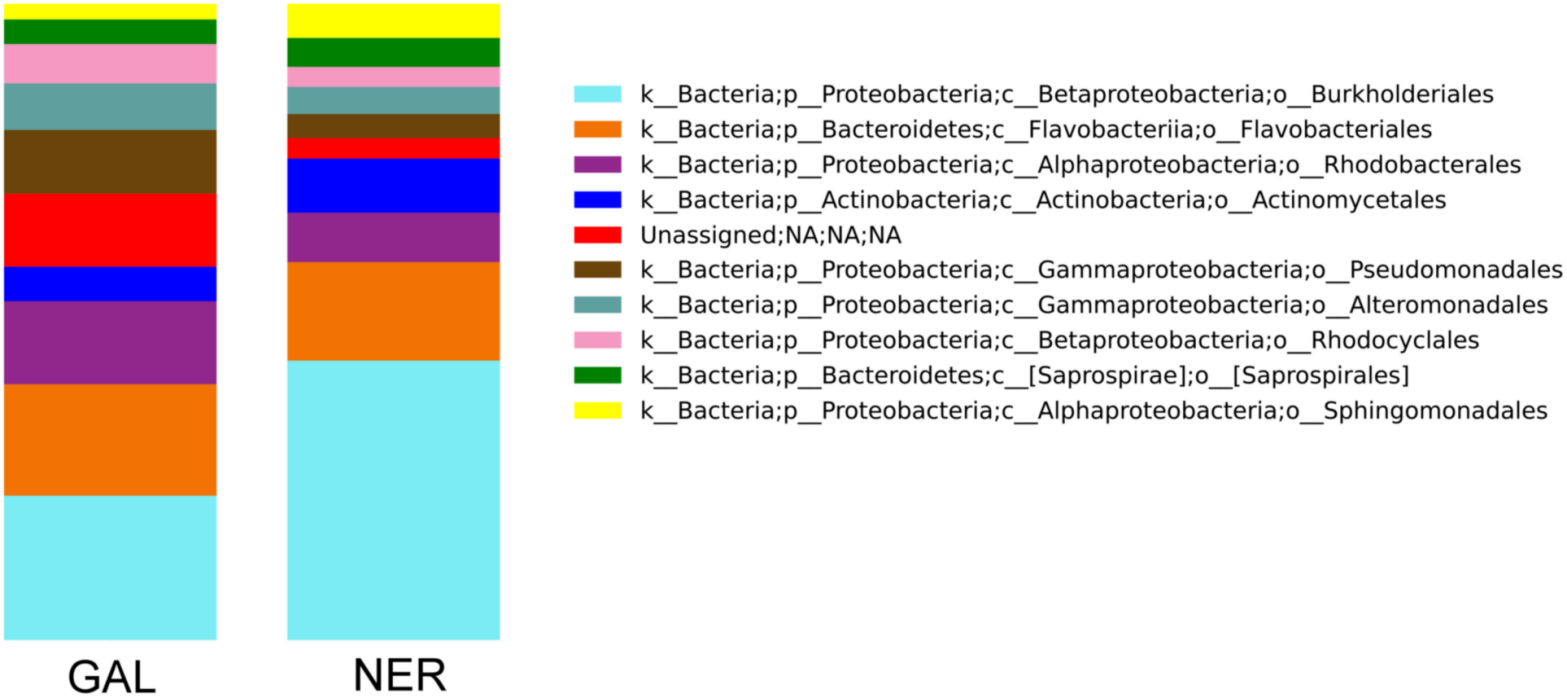
Microbial taxonomic composition of the Estuary of Bilbao tributaries Nervion (NER) and Galindo (GAL). In the bar-plot, each column shows the mean relative abundances of the top 10 most abundant orders in each tributary in April, August, and October 2014. The taxonomic groups represented in the plot account for 63% of the total community.

A distinguishable bacteria profile was found in the main tributaries of the Estuary of Bilbao (Fig 6). The community of the tributary with the highest water discharge (66%), the Nervion (NER), was dominated by *Burkholderiales*, which represents up to 32% of the community. Regarding the Galindo tributary (GAL), the most abundant orders were the following: *Burkholderiales* (11.92%), *Flavobacteriales* (9.2%), *Rhodobacterales* (6.86%), and *Pseudomonadales* (5.27%). These samples were collected 5 meters downstream from a WWTP outlet and showed a unique composition of OTUs related to *Sulfurimonas*, *Bdellovibrio*, and *Zoogloea* genus (S5 Table).

**Fig 7:**
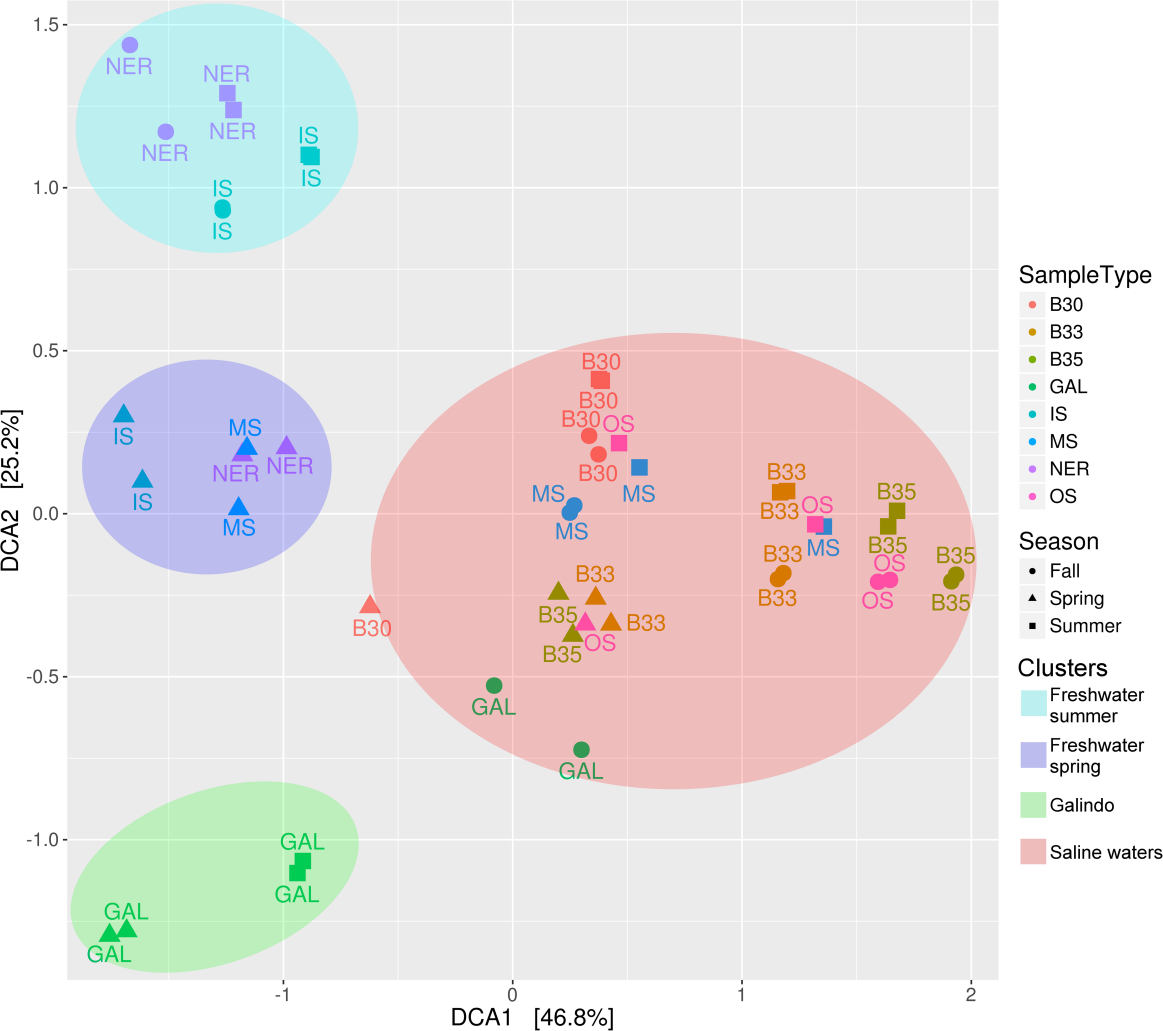
Bacterial community distribution for Estuary and tributary samples. A Bray-Curtis DCA plot showing the community dissimilarity among estuarine water samples (IS, MS, OS, B30, B33, B35) and the samples of the two tributary stations (GAL, NER) collected in April, August, and October 2014.

When analyzing the samples from the Estuary of Bilbao together with the two tributaries, the communities were grouped first by salinity and then by season (Fig 7), driven by precipitation and temperature changes. Freshwater masses (IS, NER, GAL) were further divided into three clusters: A) Galindo samples’ community that clustered apart, B) freshwater spring and C) freshwater summer (Fig 7). However, Galindo's samples during fall were influenced by brackish water (salinity = 22 ppt) and so those bacterial communities clustered with saline water samples.

## Discussion

Expanding upon the knowledge about bacterial community´s cycles in highly dynamic ecosystems, such as estuaries under the influence of the mixed effect of tides, strong salinity gradients, and sporadic flood events, should lead to a better understanding of the adaptability of microorganisms to different physicochemical changes [3,8,12]. Furthermore, regular monitoring of bacterial diversity changes along environmental and pollution gradients would distinguish factors that influence estuarine ecosystem variation. Recent developments in DNA sequencing techniques allow screening taxonomic diversity in water samples affordably and reliably [46]. In this regard, by applying 16S rRNA amplicon sequencing to different salinity water masses along an annual cycle, this study is the first detailed survey of bacterial diversity in the Estuary of Bilbao. The main tributaries were included in the study and several environmental parameters (such as salinity, temperature, dissolved oxygen concentration, turbidity, pH, and chlorophyll concentration) were observed to give further context to the revealed patterns. High rainfall during the winter and spring, a period of low DO concentration with high levels of chlorophyll and temperature in the inner estuary (B30) in summer [13,15,21,25], and the thermal variation happening from winter to summer—all appeared to correlate with fluctuations in the bacterial community. Despite these various microbial compositions, a similar community makeup was found at both the beginning and end of this yearlong study, suggesting an annual cycling of the microbial community.

### The Estuary of Bilbao: The case of a small urban estuary

The geomorphology of the Estuary of Bilbao contrasts to that of large estuaries characterized to date through amplicon sequencing (Mississippi [19], Sydney [20], etc.). The Estuary of Bilbao is short (~20 km) with low river discharge (max = 37 m^3^ s^-1^) and a contrasting tidal fluxing rate (max = 700 m^3^ s^-1^ in the outer zone and max = 10 m^3^ s^-1^ in the inner zone) [13]. Together, these factors cause bacteria to have a short residence time in surface waters. However, similar to the results shown in large estuaries [7,19,20], the bacterial communities of the Estuary of Bilbao were classified in two large groups according to salinity: brackish-euhaline communities (salinity > 5 ppt) and freshwater communities (salinity < 5 ppt) (Fig 4). Accordingly, the Spearman correlation values between OTUs and environmental features showed highest rho values when testing for salinity (rho = 0.72–0.85 for salinity and rho = 0.52–0.64 for temperature, S2 and S4 Tables). Further, within the groupings (brackish-euhaline and freshwater), samples clustered by season (Fig 4A), revealing temperature as a secondary driver of community drifts. This is in line with previous work showing that aside from salinity, temperature significantly influences the microbiomes of long and short estuaries, as well as estuaries under high or low anthropogenic pressures [2,4,6,20].

Outside seasonal variation, the euhaline water samples (B30, B33, B35) had a relatively stable core microbial community due to the ocean’s buffer effect. These results are consistent with Chow and colleagues (2013) findings that reported little oscillation in the communities of the upper ocean layers [47]. Contrarily, estuarine surface layers waters, particularly MS and OS (Table 1 and S1 Table), showed higher variability in both environmental features and bacterial community structure (Fig 4). Sugimoto and colleagues [48] found that in the surface waters of Ise bay in Japan, salinity and other environmental parameters (turbidity, temperature, etc.) were significantly altered by the river discharge and precipitation. Similarly in the Estuary of Bilbao, MS and OS samples become brackish during rainy periods (Fig 2), showing a high number of freshwater-related taxa (e.g. *Burkholderiales*, S2 File) as well as an increase in their alpha diversity (Fig 2). Accordingly, brackish waters have been considered among the richest waters in the estuary [1,7,49–52]).

In high precipitation periods, the river discharge increases and curtails the residence time of the water [13] encouraging the presence of freshwater-related taxa in the outer waters of the Estuary of Bilbao, where flow is lessened (e.g. *Burkholderiales*, S2 File). Moreover, an increase of alpha diversity was observed during the high precipitation period (Fig 2) in the surface waters of the lower half of the estuary (MS and OS). Similar results have been shown in previous studies (e.g. [1,7,49–52]) where brackish waters have been noted among the richest portions of an estuary with high bacterial diversity. Assumedly, precipitation and river discharge affect an estuary’s brackish waters, where mixed communities comprised of bacterioplankton populations from multiple water masses are able to interact [52].

Apart from these general patterns, summer eutrophication events have been reported since the 19th century in the inner part of the urban Estuary of Bilbao following water stratification [13,15,22,26]. Similarly, summer B30 samples from this study were characterized by a severely low DO concentration with high temperatures and chlorophyll concentrations (Table 1 and S1 Table). This situation is believed to be a consequence of the conversion of the original estuary by the large-scale reclamation of intertidal areas into a minor tidal channel [14]. As outlined by Uriarte and colleagues [13], the channelization causes an increase in water turnover time of the inner euhaline water, leading to a decrease in DO concentration that coincides with the decrease of tributary water discharge. This particular environmental situation leads to a distinct community in summer B30 euhaline samples. For instance, OTUs related to *Candidatus Aquiluna rubra* stood out as the ones with highest abundance increases during low DO concentrations. This species has been previously described and detected in eutrophic freshwater [53] and in harbors’ seawater [54]. Additionally, *Comamonadaceae* family and *Flavobacterium* genus increased in abundance one month before the decrease in DO concentration (Fig 5). Although these bacteria are known freshwater-typical aerobic organisms, they can become facultative anaerobic denitrifiers [55,56]. Thus, they could survive in anoxic waters as they perform denitrification. Several variables are interrelated in eutrophication processes [13, 15, 22, 26] (e.g. DO is negatively correlated to temperature, while the correlation between chlorophyll concentration and temperature is positive), which is evidenced further in this community (Fig 5). Additional environmental factors that were not measured in this study, such as nutrients, wind, nitrites, nitrates, etc., may play a significant role in defining the microbial community and would be necessary measures to construct a higher resolution interaction network. The sequencing of an additional gene would also help in a better understanding of the OTUs and environmental factors’ interactions, as it would allow a better taxonomic categorization of OTUs.

### Tributaries

Similarly to the estuarine communities, the bacteria communities of the tributaries were grouped by salinity (Fig 7). However, both tributaries waters (Galindo and Nervion-Ibaizabal) each had a distinguishable community and specific physicochemical properties that characterized them.

Galindo tributary samples, which were collected downstream of a WWTP, were the most unrelated samples due to the bacteria used in the activated sludge. Within them, some OTUs belonging to *Bdellovibrio* and *Zoogloea* genera stood out. These types of bacteria proliferate in the different steps of wastewater treatment [57] and they are related with different processes of water treatment (e.g. *Bdellovibrio* as a bacterial predator). Furthermore, *Zoogloea* species are typically used in domestic and aerobic sewage-treatment systems, such as trickling filters, activated sludge tanks, or oxidation ponds [57]. In light of this, we can conclude that since the WWTP implementing a biological treatment step in 2001–2002, which dramatically reduces the contribution of the plant effluent to the river [58], the water discharge contains bacteria from the activated sludge. However, as amplicon sequencing does not distinguish between living and dead cells, it remains to be tested whether these bacteria are functionally active; thus, a shotgun or metatranscriptomics approach would be needed. In any case, these taxa were not detected in the estuarine samples (as can be seen in S5 Table), meaning that either: 1) they get diluted in the estuarine waters and a higher sequencing coverage might be needed to identify them; or 2) the low river discharge of this tributary might not be enough to counter the tidal flux of the estuary and freshwater mass would shift upwards when the tide rises [59], making the waters stagnant in the inner Galindo's basin.

Regarding the Nervion tributary, *Comamonadaceae—*related OTUs were the most abundant family (27.3±7.5% of the bacterial community), while its abundance only represented 4.43±6.37% in outer estuarine waters (B35) (S2 File). Interestingly, *Comamonadaceae* abundances found in the Estuary of Bilbao are among the highest detected compared to other studied estuaries [7,60,61]. Within this family, the most abundant two OTUs could not be classified beyond the family taxonomic level, as shown in Fig 5. *Comamonadaceae* has a remarkable metabolic diversity that includes aerobic organotrophs, anaerobic denitrifiers and Fe^3+^-reducing bacteria, hydrogen oxidizers, photoautotrophic and photoheterotrophic bacteria, and fermentative bacteria [62,63].

In conclusion, in this preliminary survey of the bacterial diversity of the Estuary of Bilbao, the sequencing of the 16S rRNA gene showed that salinity and temperature are the most prominent abiotic factors varied synchronously with bacterial community changes in this estuary, as is the case with larger estuaries. Moreover, additional environmental factors need to be studied to acquire a more representative picture of the dynamics of the estuary’s diverse water masses. For instance, precipitation and resulting river discharge is linked to the appearance of mixed communities in surface waters. Additionally, certain OTUs correlated with DO concentration, temperature, and chlorophyll concentration in the inner euhaline waters in summer expose a unique community characterized by a higher abundance of facultative anaerobic denitrifiers. The defining characteristics of each river (orography, stratigraphy, different types of anthropogenic impact) contribute different substrates to the bacterial communities of the estuary and therefore, future studies addressing these factors are recommended. Future endeavors would involve sampling a more expansive area of each river/tributary to be able to characterize the provenance of each bacterial assemblage and to indirectly monitor discharges from the different anthropogenic sources (human and industrial waste, WWTP, etc.) potentially affecting the system. Furthermore, studying the metabolic cycles of these communities via gene expression analysis (i.e. metatranscriptomics; [64]) would give further insights into the biochemical dynamics beyond taxonomy. Thereby, functional metagenomic research could improve our understanding of bacterial functions in specific biochemical cycles related to anthropogenic pressure (e.g. sulfur-reduction and denitrification processes) in these ecosystems.

## Supporting information

S1 Table Physico-chemical values for each estuarine water mass by sampling date. **Water** samples were collected monthly between August 2013—Sept. 2014 except when weather precluded it.
(CSV)

S2 Table Bacterial families significantly correlated with salinity. The top 5 most significant bacterial families showing either negative or positive Spearman correlations to salinity (bonferroni p value <0.01).
(CSV)

S3 Table Spearman's correlation values between environmental variables and bacterial community. Spearman's values for temperature, salinity, precipitation and dissolved oxygen (DO), for each water mass.
(CSV)

S4 Table Bacterial families significantly correlated with temperature. The top 5 most significant bacterial families showing negative or positive Spearman correlations with temperature (bonferroni p value <0.01).
(CSV)

S5 Table Unique OTUs in Galindo tributary. OTUs that were significantly more abundant in Galindo river (Kruskal-Wallis, FDR value <0.01) and that were not present in the rest of estuaries/tributaries (and thus were unique for Galindo tributary) are shown in the table.
(CSV)

S1 File Within environmental variables correlations per water mass. Principal Components Analysis (PCA) plots for samples (“individual factor map”) and environmental variables distribution (“variables factor map”).
(DOC)

S2 File Community changes along the annual cycle per estuarine water mass. Taxonomy barplots show orders with greater abundance than 1%. The labels indicate the collection date of each sample.
(DOC)

S3 File Core-OTUs per water mass. A) Venn diagram Classification showing the per water mass core-OTUs, defined as OTUs present in 100% of samples throughout the year. B) Core-OTUs presence in the different water masses: The first column shows the taxonomic classification for each core-OTU, the second column indicate the type of distribution of each core-OTU (ubiquitous, pan or unique) and the third column indicate the water masses where the core-OTU were found.
(DOC)
