# Supplementary figures and images for: Unraveling the environmental and anthropogenic drivers of bacterial community changes in the Estuary of Bilbao and its tributaries
Source: PLoS One. 2017 Jun 8;12(6):e0178755. doi: 10.1371/journal.pone.0178755 (PMC5464593; doi:10.1371/journal.pone.0178755)

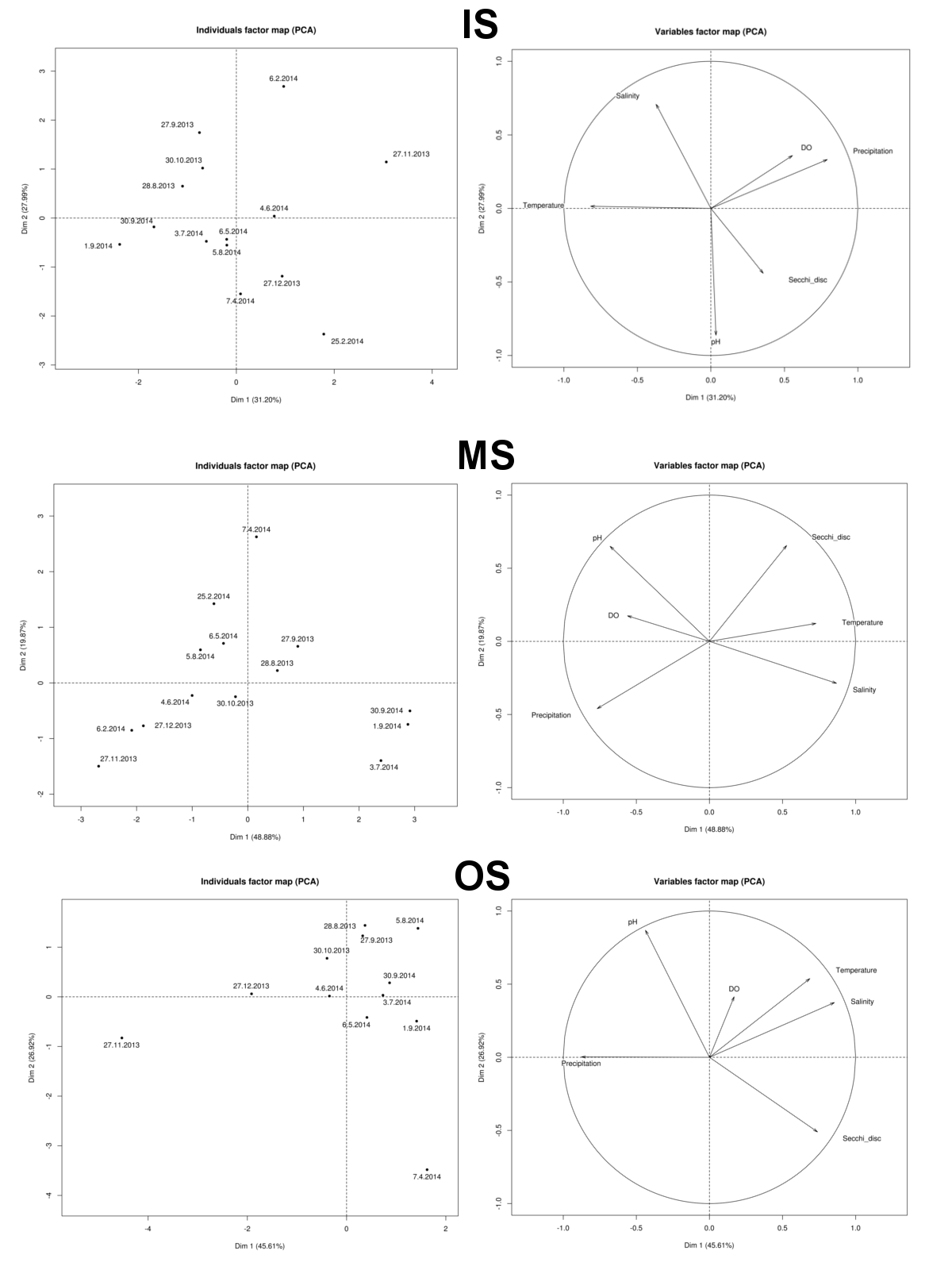


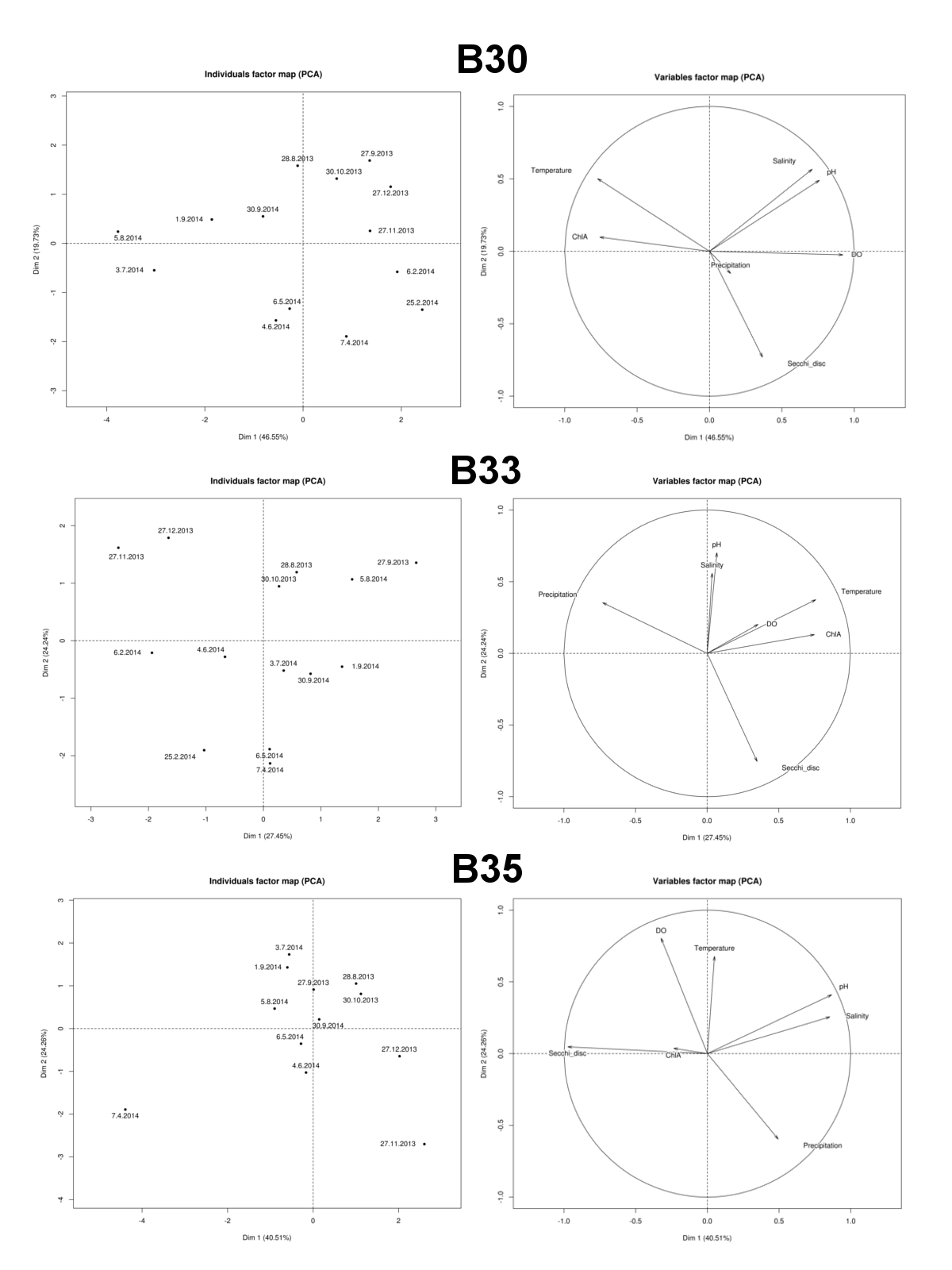

Supplement: S1 File — Principal Components Analysis (PCA) plots for samples (“individual factor map”) and environmental variables distribution (“variables factor map”). (DOC) [file pone.0178755.s006.doc]

IS water mass


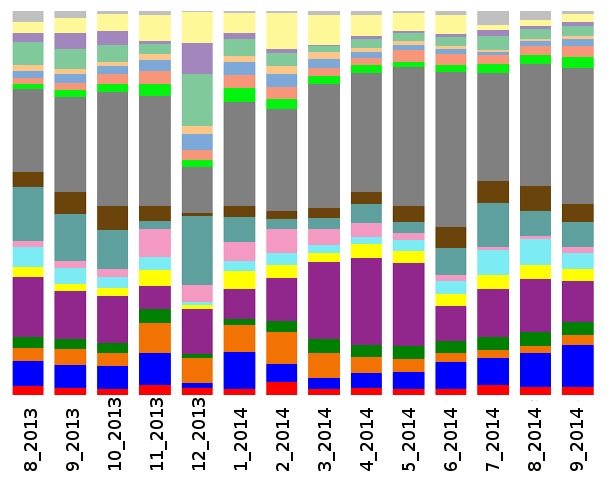


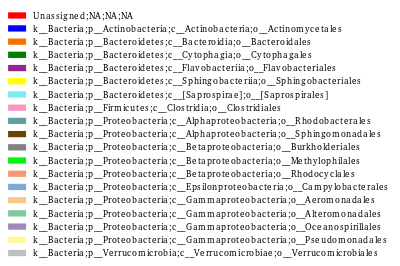


MS water mass


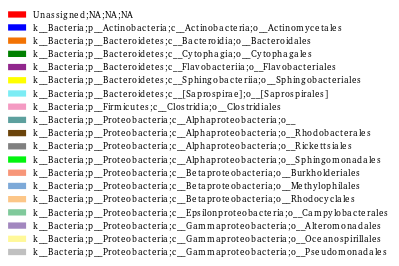

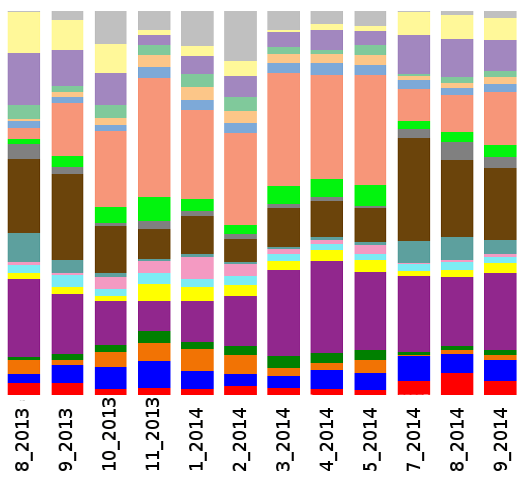


OS water mass


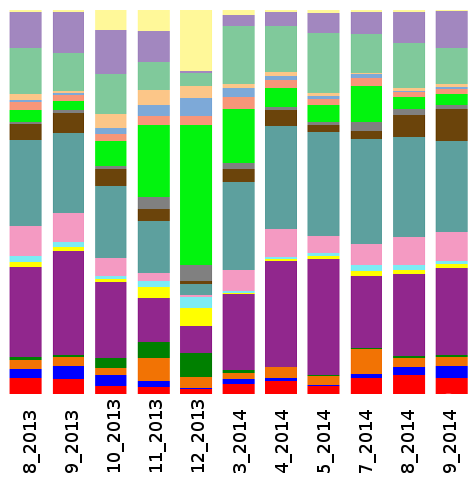


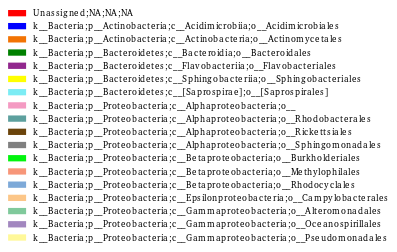


B30 water mass


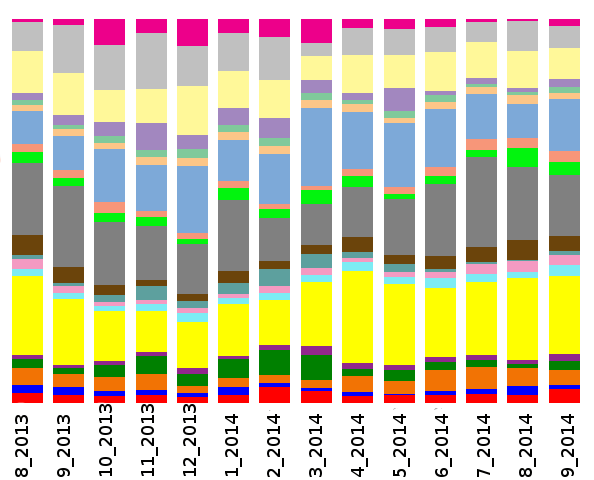


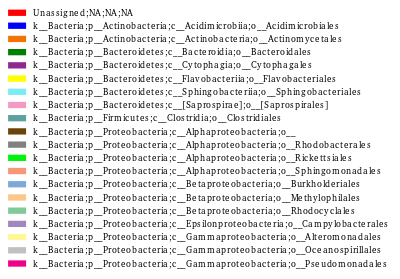


B33 water mass


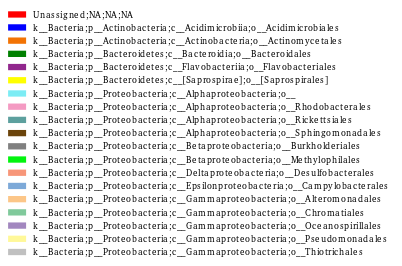

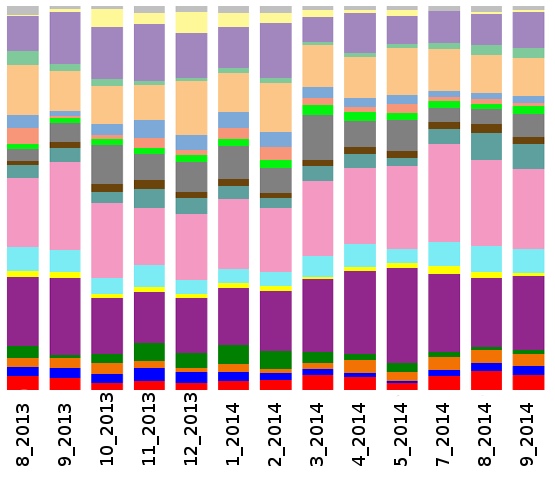


B35 water mass


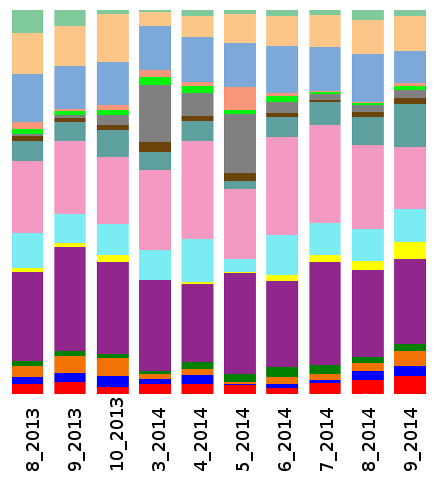


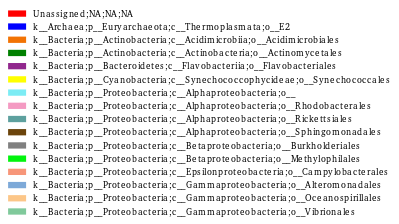

Supplement: S2 File — Taxonomy barplots show orders with greater abundance than 1%. The labels indicate the collection date of each sample. (DOC) [file pone.0178755.s007.doc]
