# Supplementary material for: Unraveling the environmental and anthropogenic drivers of bacterial community changes in the Estuary of Bilbao and its tributaries
Source: PLoS One. 2017 Jun 8;12(6):e0178755. doi: 10.1371/journal.pone.0178755 (PMC5464593; doi:10.1371/journal.pone.0178755)
Supplement: S3 File — A) Venn diagram Classification showing the per water mass core-OTUs, defined as OTUs present in 100% of samples throughout the year. B) Core-OTUs presence in the different water masses: The first column shows the taxonomic classification for each core-OTU, the second column indicate the type of distribution of each core-OTU (ubiquitous, pan or unique) and the third column indicate the water masses where the core-OTU were found. (DOC) [file pone.0178755.s008.doc]

A) Venn diagram Classification for each water mass' core-OTU, at 100%, throughout the year.


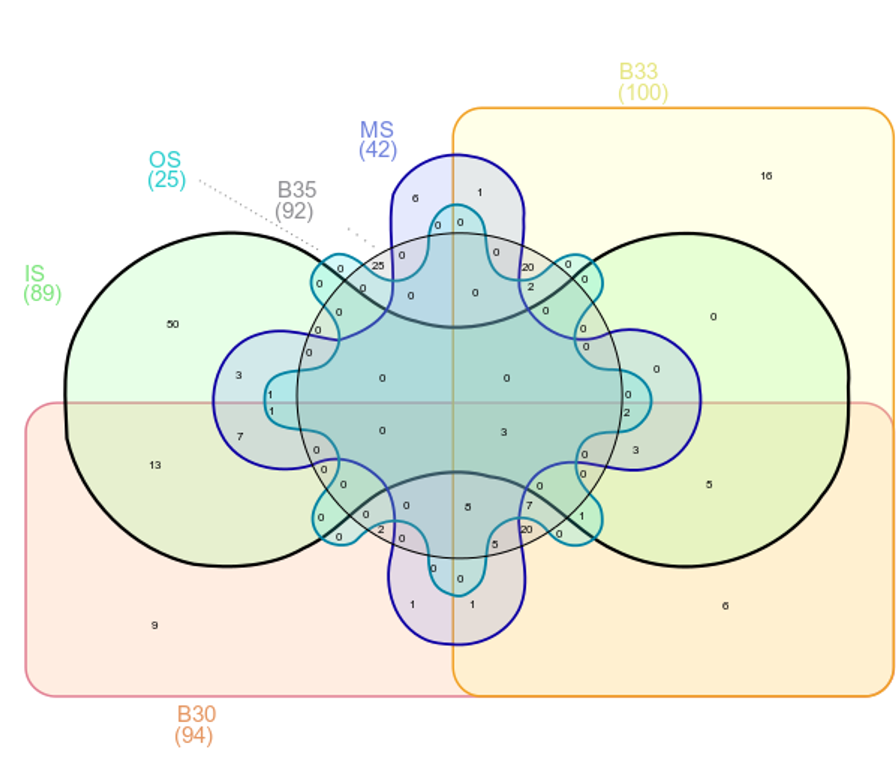


B) A table with the core-OTUs distribution between the different water masses: The first column shows water masses that share the OTUs, the second column indicate the number of shared OTUs and in the third are the taxonomy of the OTUs.
